# Genome-wide association analysis of adaptation to oxygen stress in Nile tilapia (*Oreochromis niloticus*)

**DOI:** 10.1186/s12864-021-07486-5

**Published:** 2021-06-09

**Authors:** Xiaofei Yu, Hendrik-Jan Megens, Samuel Bekele Mengistu, John W. M. Bastiaansen, Han A. Mulder, John A. H. Benzie, Martien A. M. Groenen, Hans Komen

**Affiliations:** 1 Wageningen University & Research, Animal Breeding and Genomics, Wageningen, The Netherlands; 2 School of Animal and Range Sciences, College of Agriculture, Hawassa University, Hawassa, Ethiopia; 3 WorldFish Centre, Jalan Batu Maung, Bayan Lepas, Penang Malaysia; 4 School of Biological Earth and Environmental Sciences, University College Cork, Cork, Ireland

**Keywords:** Nile tilapia, Growth, Hypoxia, Oxygen stress, GWAS, Meta-analysis

## Abstract

**Background:**

Tilapia is one of the most abundant species in aquaculture. Hypoxia is known to depress growth rate, but the genetic mechanism by which this occurs is unknown. In this study, two groups consisting of 3140 fish that were raised in either aerated (normoxia) or non-aerated pond (nocturnal hypoxia). During grow out, fish were sampled five times to determine individual body weight (BW) gains. We applied a genome-wide association study to identify SNPs and genes associated with the hypoxic and normoxic environments in the 16th generation of a Genetically Improved Farmed Tilapia population.

**Results:**

In the hypoxic environment, 36 SNPs associated with at least one of the five body weight measurements (BW1 till BW5), of which six, located between 19.48 Mb and 21.04 Mb on Linkage group (LG) 8, were significant for body weight in the early growth stage (BW1 to BW2). Further significant associations were found for BW in the later growth stage (BW3 to BW5), located on LG1 and LG8. Analysis of genes within the candidate genomic region suggested that MAPK and VEGF signalling were significantly involved in the later growth stage under the hypoxic environment. Well-known hypoxia-regulated genes such as *igf1rb*, *rora*, *efna3* and *aurk* were also associated with growth in the later stage in the hypoxic environment. Conversely, 13 linkage groups containing 29 unique significant and suggestive SNPs were found across the whole growth period under the normoxic environment. A meta-analysis showed that 33 SNPs were significantly associated with BW across the two environments, indicating a shared effect independent of hypoxic or normoxic environment. Functional pathways were involved in nervous system development and organ growth in the early stage, and oocyte maturation in the later stage.

**Conclusions:**

There are clear genotype-growth associations in both normoxic and hypoxic environments, although genome architecture involved changed over the growing period, indicating a transition in metabolism along the way. The involvement of pathways important in hypoxia especially at the later growth stage indicates a genotype-by-environment interaction, in which MAPK and VEGF signalling are important components.

**Supplementary Information:**

The online version contains supplementary material available at 10.1186/s12864-021-07486-5.

## Background

Tilapia is one of the most important species in aquaculture noted for their relative ease of culture and rapid growth. Tilapia is currently cultured in over 120 countries, mainly in the tropics and sub-tropics, with a production from 0.3 million tonnes in 1987 to closely 7 million tonnes in 2018, which makes it the second largest aquaculture species in the world [1]. Tilapia is a valuable protein source in developing and emerging economies. Due to its wide range of culturing conditions, tilapia is also an excellent model to study adaptive responses to environmental stresses [2]. One of the most important non-commercial breeding programs is the Genetically Improved Farmed Tilapia (GIFT), executed by WorldFish in Malaysia. It has sustained genetic gains for growth and body trait more than 10% per generation for more than six generations [3]. However, rapid growth potentially exacerbates existing limitations in the production environment. In non-aerated ponds, high stocking density can lead to an extreme hypoxic environment, especially at the end of the night (nocturnal hypoxia), when algae have higher rate of oxygen consumption than oxygen production. The extreme hypoxic environment can lead to lower feed intake, stagnated growth, and susceptibility to disease [4, 5]. The result is a higher mortality and lower yield than what could potentially be achieved [6]. The effects can be mitigated through mechanical aeration of ponds, but a daily fluctuation in oxygen availability is nevertheless inevitable.

Response to hypoxia is a highly complicated biological process that has received considerable scientific attention, both in fishes and in land vertebrates (e.g. high-altitude adaptation studies). Most of these response processes happen very early at the onset of hypoxia through the activation of pathways depending on proteins that are already present [7]. But in the longer term, adaptive responses to hypoxia are leading to different expression of genes. In mammals, studies in the past decades pointed to an essential role of the Hypoxia-inducible factors (HIF) for gene expression regulation during hypoxia [8]. Other genes such as tyrosine hydroxylase (TH), phosphoglycerate kinase 1 (PGK1) and vascular endothelial growth factor (VEGF) are also important key actors [9]. Recent studies have described that fish have homologs of HIF-α and -β, which may show similar function to those in mammals in the hypoxic environment [9, 10]. Several other hypoxia-related proteins and signal pathways have been reported, such as AMP-activated protein kinase (AMPK), reactive oxygen species (ROS), mitogen-activated protein kinase (MAPK) and IGF-1/PI3K/AKT signalling, which have been reported to to be involved in hypoxia adaptation of some fish species [11, 12].

Genetic adaptation to hypoxia is important for survival in many aquatic species, since variation in oxygen availability in water can vary far more, and far more rapidly, than in terrestrial ecosystems. Hypoxia is an important cause of economic losses in aquaculture. Understanding the genomic architecture of hypoxia adaptation could help to improve resilience through breeding programs for economically important species. So far, hypoxia tolerance has been studied in a limited number of fish species, including catfish [13, 14], Atlantic salmon [15], and tilapia [16], with the aim to identify QTLs for hypoxia-tolerant traits. Genome-wide association study (GWAS) has been regarded as a powerful tool to identify genetic markers associated with target traits, and a more complete gene network will provide the knowledge bases required for the aquaculture industry to make improvements [17]. In hybrid catfish, Zhong et al. [13] revealed in total nine SNPs associated with dissolved oxygen (DO) level using a 250 K SNP array. Analysis of the genes overlapping or close to those SNPs suggested that many of those genes were involved in the PI3K/AKT and VEGF pathways. In another study, Brennan et al. [18] aimed to identify population differences in hypoxia tolerance by calculating the amount of time for Killifish to lose equilibrium using GWAS. They found that variation in Hyaluronan synthase 1 (*has1*) influenced the production of hyaluronan, which can directly effect on hypoxia tolerance.

There are only a few studies that focused on genetic bases of either hypoxia tolerance or growth in Nile tilapia [16, 19], however, none of these investigated how hypoxia influences growth in Nile tilapia. The main objective of this study was to unravel the genomic architecture associated with phenotypic variation during adaptation to hypoxia or normoxia, and to elucidate the effect of hypoxia on the genetic regulation of growth.

## Results

### Phenotype statistics

Fish fry was produced from generation 15 of the GIFT breeding program. The experiment was carried out in an aerated (normoxic) and non-aerated (nocturnal hypoxic) ponds, each producing 1026 and 1037 fish that were involved in the analysis. Body weight of growing fish was measured at five time points (Table 1). The data show that the number of tilapia in both environments gradually decreased. This effect was more pronounced in the hypoxic environment, with a total loss from stocking to harvest of 23% of the initial number of individuals, compared to 14% in the normoxic environment. The average body weight at five time points in the normoxic environment was significantly higher than those in the hypoxic environment, with the exception of the first time point (BW1). Interestingly, the coefficient of variation in body weight (CV) at each time point in the two separate environments decreased.

**Table 1:** Summary statistics of body weight across the whole growth period in Nile tilapia

Trait	Days	Environments	No.	Mean	Max	Min	SD	CV(%)	***P*** value
BW1	0	Hypoxia	1037	24.8	77.0	3.6	13.4	54.0	0.14
	0	Normoxia	1026	25.4	77.1	2.9	13.1	51.7	
BW2	55	Hypoxia	1037	144.3	328.0	26.0	54.7	37.9	3.81E-07
	56	Normoxia	1026	159.1	394.3	30.2	63.1	39.7	
BW3	104	Hypoxia	907	265.9	498.3	70.5	73.3	27.6	4.17E-08
	105	Normoxia	941	289.4	650.5	63.3	92.5	32.0	
BW4	167	Hypoxia	885	426.4	805.3	117.0	118.9	27.9	2.20E-16
	168	Normoxia	903	533.6	1079.1	68.2	177.2	33.2	
BW5	217	Hypoxia	799	579.6	1003.4	135.5	154.4	26.6	2.20E-16
	218	Normoxia	885	780.9	1588.6	185.7	265.6	34.0	

*BW* Body weight, days means the growing out days in either hypoxia or normoxia, *No.* The number of animals, *Max* Maximum, *Min* Minimum, *SD* Standard deviation, *CV%* Coefficient of variation

The estimated phenotypic correlations for body weight between different time points in the two environments are shown in Table 2. Results show that phenotypic correlation between time points in the hypoxic and nomorxic environments was initially high (0.80 and 0.81 separately), but decreased with increasing time between measurements.

**Table 2:** Phenotypic correlations of body weight across the whole growth period in different environments

Trait	BW1	BW2	BW3	BW4	BW5
BW1	–	0.81	0.61	0.32	0.22
BW2	0.80	–	0.77	0.44	0.32
BW3	0.59	0.80	–	0.66	0.52
BW4	0.29	0.46	0.68	–	0.83
BW5	0.15	0.31	0.56	0.85	–

The spearman’s rank correlation coefficient of body weight in hypoxia is presented below diagonal, while the normoxia is above diagonal

### SNP statistic and population structure

**Fig. 1:**
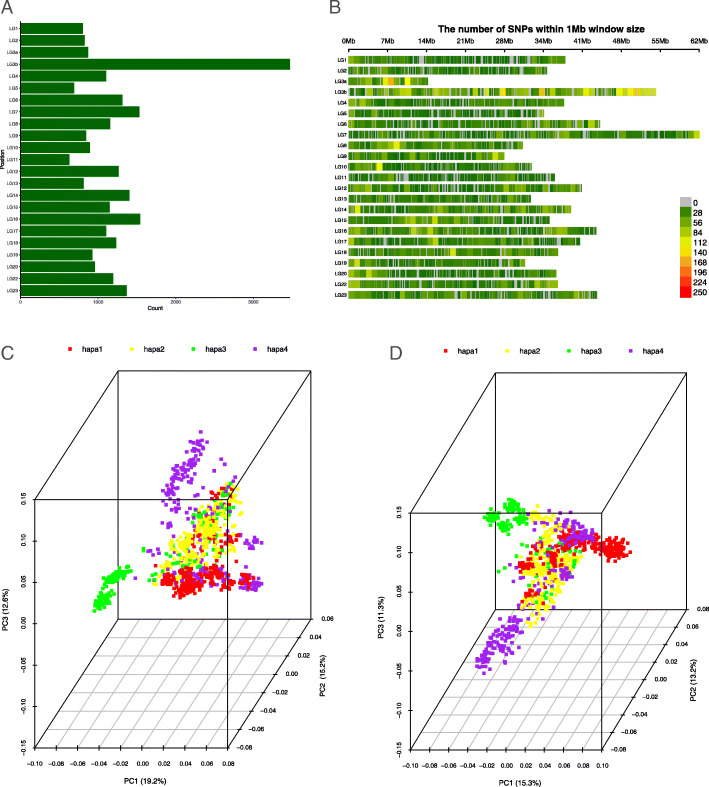
SNP statistics with all individuals. **a** Histogram of SNPs distribution across all linkage groups. **b** SNP density plots across all linkage groups. **c** and **d** 3D PC plot for origin of tilapia at BW1 in the hypoxic (**c**) and normoxic (**d**) environments using all SNPs that passed filtering, where each dot represents one individual

In total 27,090 SNPs that passed SNP minor allele frequency, genotype and individuate call rate criteria, were used for subsequent analysis. Those SNPs were found to be randomly distributed across the genome with a density of approximately 28 SNP per Mb. The highest number of SNPs (4344) on LG3 while LG11 had the lowest number of SNPs (630) (Fig. 1a). A few windows on LG3 show a higher density of SNPs (Fig. 1b). Besides this exception, the distribution of SNPs is uniform with the linkage group physical length of the *Oreochromis niloticus* genome (GenBank accession GCF_001858045).

The PCA represents the genetic structure for individuals from the hypoxic and normoxic environments, respectively (Fig. 1c, d and Supplementary Figures 3 and 4). In the hypoxic environment, the first three principal components (PCs) explain 47.0% of the total genotype-based variation and separate samples according to their family differences. PC1 accounts for 15.2% of the total genotype variation and separates families in hapa3 with other families. In the normoxic environment, the first three components explain 39.8% of the total genotype variation, while the first component accounts for 15.3%. Moreover, the largest PC (PC1) of all samples separates disperse cluster from families in hapa3 again.

These results indicated that there was clear genetic variation caused by family differences in both environments. This was partially caused by the different distribution of the number of fish from four rearing hapas under the normoxic and hypoxic environments. Additionally, the average body weight of fish in hapa3 was larger than that of other hapas, especially the mean body weight of male fish at the first time point was much higher in the normoxic environment than the hypoxic environment (Supplementary Figure 2), indicating that a few families with high body weight dominated in one environment but not the other.

### Single environmental GWAS at five different time points

**Fig. 2:**
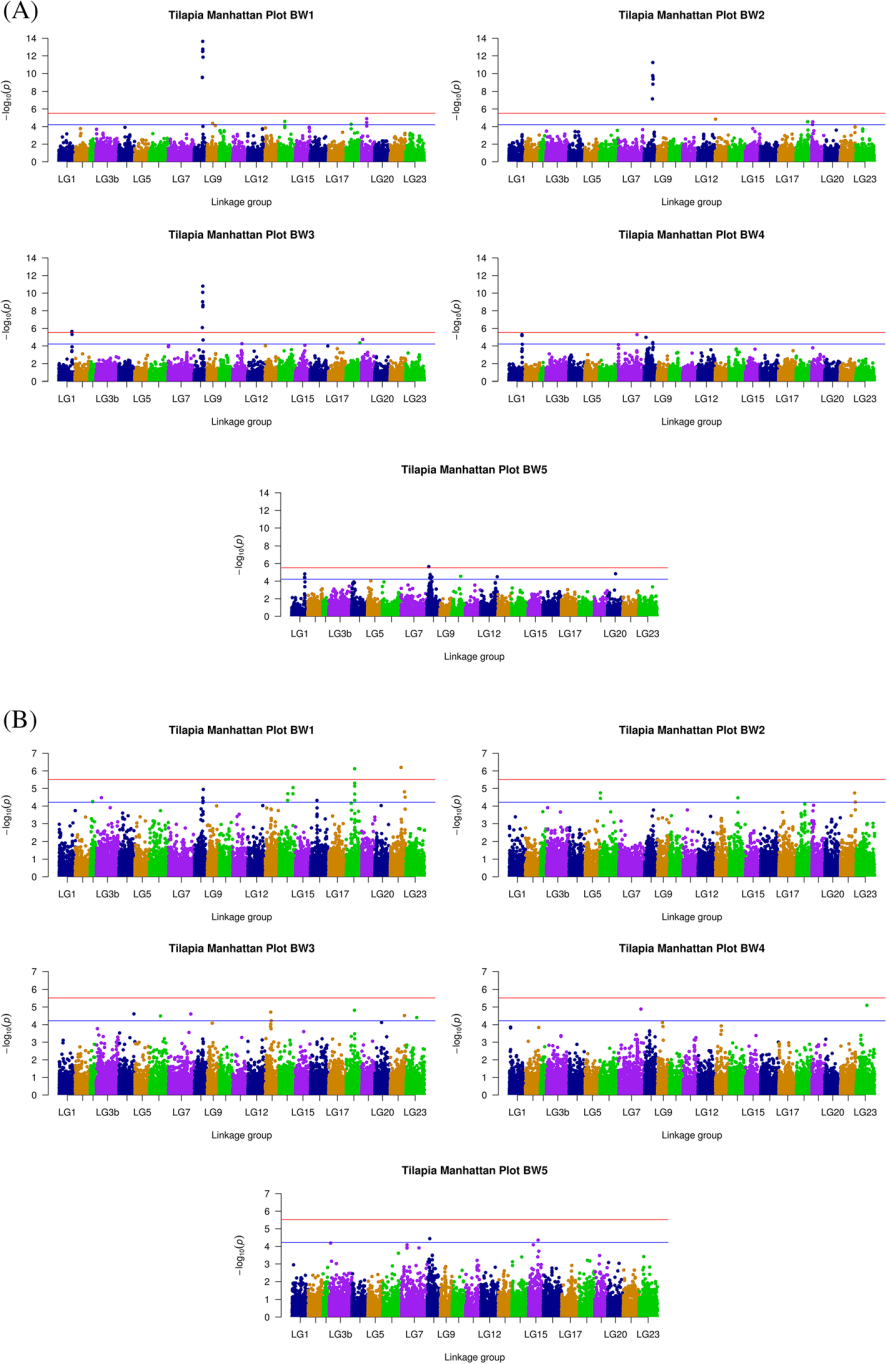
Manhattan plots across the whole growth period in the hypoxic environment. **a** and normoxic environment (**b**). Each dot on this figure corresponds to a SNP within the dataset, while the orange and blue horizontal line represent the genome-wide significance (5.52) and suggestive significance threshold value (4.22), respectively. The Manhattan plots contain –log10 observed *P*-values for genome-wide SNPs (y-axis) plotted against their corresponding position on each chromosome (x-axis)

Significant SNPs were detected with a univariate GWAS by implementing a linear mixed model. We observed that sex and hapa effects can explain part of the difference in body weight. Thus, these were treated as fixed factors in our analysis. Overall, five association analyses, one for each time point where body weight was measured, were performed for each environment. The Manhattan plots for each of the five time points in the hypoxic and normoxic environments are shown in Fig. 2a and b respectively. In addition, Quantile-Quantile plots with genomic inflation factors were created to aid in estimating the influence of population structure on single environmental GWAS (shown in Supplementary Figures 5 and 6). The *P* values of corrected thresholds for suggestive and genome-wide significant levels were 4.22 (−log_10_(1/16504)) and 5.52 (−log_10_(0.05/16504)), respectively.

In the hypoxic environment, the analyses showed 10 significant and 26 suggestive SNPs associated with BW1 to BW5 (Supplementary Table 2). Among those, six SNPs between 19.48 Mb and 21.04 Mb on LG8 attained genome-wide significance for BW1 to BW3. However, those SNPs were not significant for BW4 and BW5. Two SNPs (LG1: 30766342 and LG1:30766336) were significantly associated with BW3 to BW5. Additionally, 16 SNPs above the suggestive level as defined above for BW1 to BW2 were found on LG8, LG18 and LG19, while 18 SNPs mostly located on LG1 and LG8, were found for BW4 to BW5. Interestingly, at BW3, SNPs on LG8 overlapped with BW1 and BW2, while SNPs on LG1 overlapped with BW4 and BW5, further confirming that there is a transition in genomic architecture associated with growth over time.

We also detected 2 significant and 27 suggestive SNPs across different growth stages in the normoxic environment (Supplementary Table 3). The suggestive peak at BW1 covered the same genomic region as that found for the hypoxic environment between 19.48 to 21.03 Mb on LG8. However, similar to the hypoxic environment, the significance of those SNPs declined from BW1 to BW3, a pattern also seen for the SNPs located on LG18 and LG22. A few SNPs on LG7 and LG15 also showed a signal near the suggestive level from BW3 to BW5, which could be potentially interesting, although they did not attain statistical significance.

### Meta-analysis GWAS across two environments

**Fig. 3:**
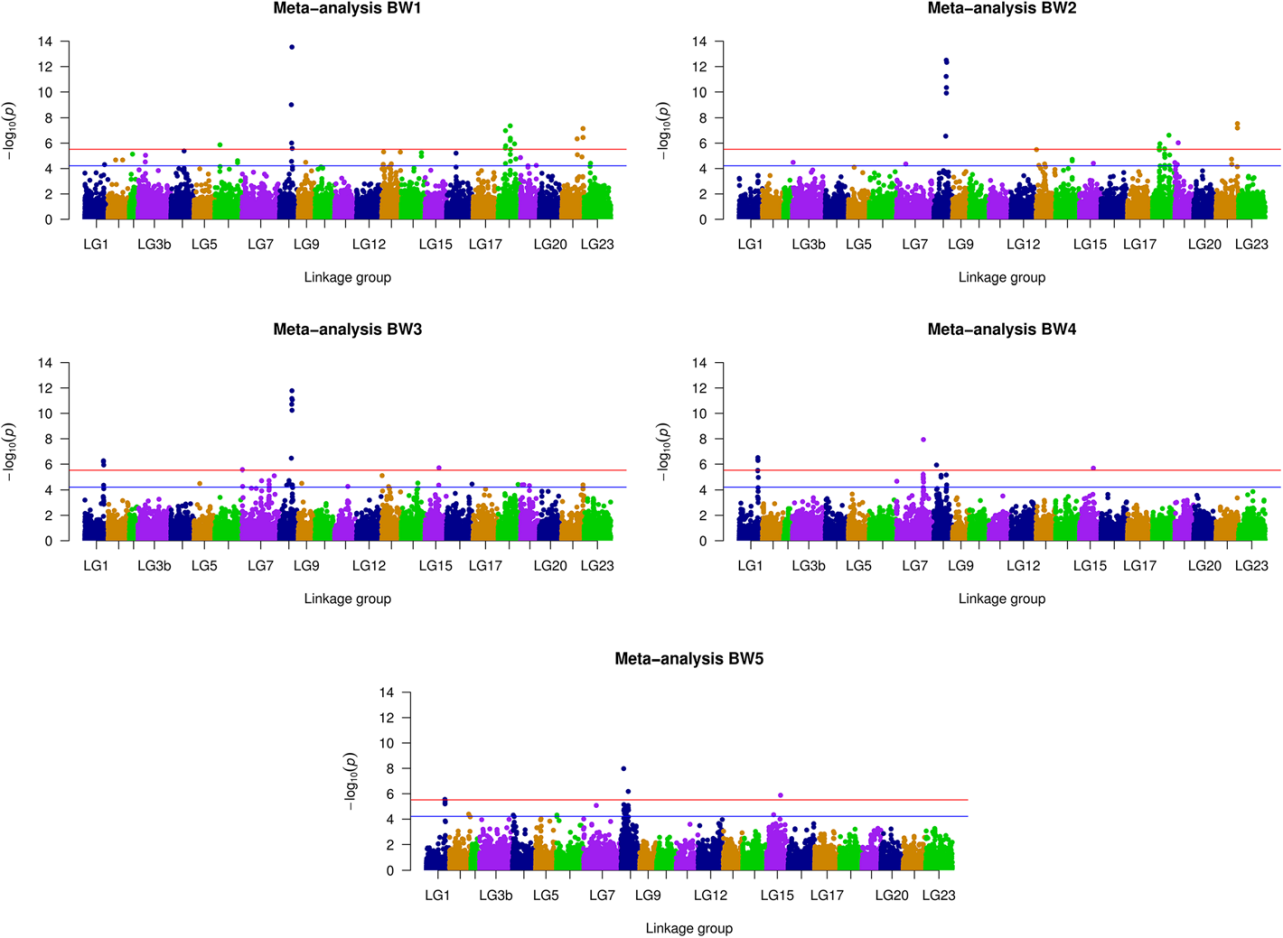
Manhattan plots of Meta-analysis GWAS across two environments. The orange and blue horizontal line represent the genome-wide significance (3.03E-06) and suggestive significance threshold value (6.06E-05) respectively

A meta-analysis GWAS that considered the effects of 27,090 SNPs in common in the hypoxic and normoxic environments was performed, and the results are shown in Fig. 3. In total 33 SNPs were detected to be significant with five measurements of body weight during the whole growth stage. Clusters of significant SNPs were mostly found on LG8, LG18 and LG22 (Supplementary Table 4). Interestingly, six SNPs located between 19.48 and 21.03 Mb on LG8, three SNPs between 12.44 and 27.32 Mb on LG18 and three SNPs within 1 kb at 35.25 Mb on LG22, were all significantly associated with body weight at time points BW1 and BW2. However, the *P*-values of those SNPs decreased in subsequent growth periods. Five SNPs between 30.54 and 31.19 Mb on LG1, and one SNP on LG15 (LG15:23051993), were associated with body weight from BW3 to BW5. Moreover, two SNPs on LG8 (LG8:4319661, LG8: 11800435) were significant at BW4 and BW5. Notably, those SNPs located on LG8 were found at a different region compared to SNPs on the same LG in hypoxic GWAS. Hence, associations for BW1 to BW2 were different from BW4 to BW5, although BW3 shows both overlap to early and late growth stages, which could indicate that a transition in the pathways involved occurred around this stage.

### Functional annotation analysis

**Fig. 4:**
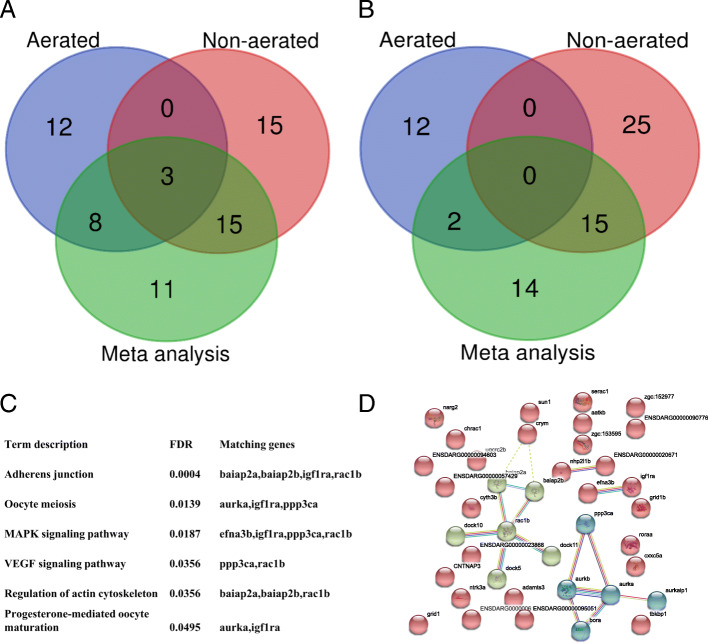
Functional annotation based on candidate genomic region associated with growth. **a** Venn diagram summarising the gene count of the early stage (BW1 to BW2) from hypoxia, normoxia and meta-analysis (cross normoxia and hypoxia). **b** Venn diagram summarising gene count of later stage (BW3 to BW5) from hypoxia, normoxia and meta-analysis. **c** KEGG enrichment of candidate genes in later stage of hypoxia environment (**d**) protein association network among candidate genes in later stage of the hypoxia environment

Based on the SNP association pattern for five measurements across the whole growth stage, we defined the early stage as BW1 and BW2, while the later stage is BW3 to BW5. Through gene identification within the associated genomic regions, the functional processes and pathways were subsequently enriched for single environmental and across environmental GWAS, respectively. Considering that BW3 is the transition point, SNPs that overlapped with the early stage were excluded in the functional annotation for the later stage. The candidate genes derived from single environment and across environment GWAS are shown in Fig. 4a and b, where 15 and 25 genes from the BW1 to BW2  and BW3 to BW5 respectively, were uniquely associated with body weight in the hypoxic environment while another 12 genes were unique to growth in the normoxic environment. It is also noteworthy that three genes (*raraa*, *rarab*, *bahcc1*) were significant for BW1 and BW2 for both single and across environmental GWAS.

During the early growth stage in the hypoxic environment, 14 GO (Gene ontology) terms were found to be significantly overrepresented (Supplementary Table 5), including central nervous system development and steroid hormone mediated signalling pathways. Six KEGG pathways were found at later growth stage (Fig. 4c), including MAPK and VEGF signalling pathways. Protein interaction network analysis showed *dock5*, *dock10*, *dock11*, *baiap2a*, *baiap2b*, *aurka* and *aurkb* strongly interacting with *rac1b* and *ppp3ca,* which all are proteins participating in MAPK and VEGF signalling (Fig. 4d).

For the early growth stage of the normoxic environment, retinoic acid receptor signalling pathway, apoptotic signalling pathway, liver development, signal transduction, steroid hormone mediated signalling pathway and brain development biological processes (Supplementary Table 6), were significantly enriched, while two (retinoic acid receptor and steroid hormone mediated signalling pathways) overlapped with the same growth period in the hypoxia environment. However, in contrast to the hypoxic environment, we did not find significant terms during the later growth stage in the normoxic environment.

In the meta-analysis GWAS across the normoxic and hypoxic environments, nine GO terms, including retinoic acid receptor signalling pathway and steroid hormone mediated signalling pathway, were mostly enriched in the early growth stage. During the later growth stage, two pathways involved in oocyte meiosis and progesterone-mediated oocyte maturation process. Interestingly, none of hypoxia-related pathway was enriched (Supplementary Table 7).

## Discussion

Hypoxia is one of the major environmental factors in fish. Hypoxia tolerance represents the ability of fish species to tolerate low oxygen level and to maintain a sustainable metabolic rate at lower dissolved oxygen levels [20]. Growth is a key trait for aquaculture and can be assessed by weight gain in order to examine the impact of hypoxic condition on fish production. For more than a half century, various and divergent claims have been made regarding the interaction between body size and hypoxia in teleost fish. Recent studies showed that small individuals have the least hypoxia tolerance within some fish species, such as Oscar cichlid [21, 22] and Red seabream [23]. In contrast, small fish chose lower oxygen levels more than large fish in Largemouth bass [24] and Yellow perch [25], however, this behaviour was suggested that the smaller fish utilize the hypoxic zone as refuge protected from the bigger predators [26]. From these studies it is clear that selection for low oxygen is difficult to ascertain, indicating a clear added value of investigations into genetic consequences of selection, such as the present study.

In general, metabolic rate is highly affected by dissolved oxygen in the rearing environment. Faster growing animals have a higher metabolic rate and therefore require more oxygen. As a consequence, hypoxia is expected to adversely affect fish growth and feed utilization [6]. On the other hand, large individuals have an obvious advantage over small ones in severe hypoxic environments because small fish will use up their glycogen reserves and reach mortality levels much faster with a higher metabolic rate [27]. Overall fish production declines, and disease resistance decreases as a consequence of hypoxia [28]. It has been observed that larger Nile tilapia tolerated low DO levels better than small ones, thought partially due to the fact that Nile tilapia immunity was stronger in larger than smaller [29]. Regardless of the complexity of the relationship between hypoxia and growth, studies focused on the genomic basis of hypoxia-growth interactions in Nile tilapia are sparse.

Our results suggest a number of genes and metabolic pathways involved in the adaptation to differences in dissolved oxygen in Nile tilapia. In the hypoxic environment, 14 significantly enriched processes were associated with the early growth stage, including nervous system development and animal organ development. *Rara* gene codes for the retinoic acid receptor alpha, a transcription factor which regulates genes involved in cellular growth and differentiation [30]. In addition, *raraa* and *rarab* play a key role during development in zebrafish [31]. Mediator of RNA polymerase II transcription subunit 24 (*med24*), an orthologue also found in human, mouse and zebrafish, participates in nervous system development [32]. However, these genes and associated molecular pathways do not indicate a clear link with hypoxia when comparing to other fish studies, and rather might reflect a relation to general growth and developmental pathways.

During the later growth stage, the results of pathway enrichment suggest that candidate regions are significantly enriched for adherens junctions, oocyte meiosis, MAPK signalling pathway, VEGF signalling pathway, regulation of actin cytoskeleton and progesterone-mediated oocyte maturation. Among these six pathways, various studies in zebrafish, channel catfish, and sea bass have shown MAPK to be involved in low oxygen tolerance in fish [14, 33, 34]. VEGF signalling was shown to be essential for maintaining the vascular density and oxygen supply in tissues [35]. Additionally, the VEGF pathway is also one of the targets of *HIF-1α*, which rapidly accumulates to activate genes involved in a series of responses to hypoxia [8, 36]. The candidate gene *igf1ra,* identified in this study, codes for IGF-1 receptor-a, a receptor of insulin-like growth factor that was reported to be a primary mediator of growth hormones [37]. The ephrin-A3 gene (*efna3*) is shown as a key functional mediator of hypoxic microenvironment and is regarded as a therapeutic target for hypoxia-specific disease [38]. Retinoic acid receptor-related orphan receptor alpha (*rora*) was demonstrated to be a key regulator of *HIF-1α* activities in human [39]. Finally, the aurora kinase A (*aurka*) gene, a serine kinase in neuroblastoma related to cell growth and migration, can up-regulate expression in human BE (2)-C cells under hypoxia [40]. Recently, Li et al. [16] also found that several regions were significantly related with hypoxia tolerance, including LG3, 4, 11, 14 and 22, especially two regions (LG4:15080000, LG11:24255000) are found to be adjacent with the peak in the hypoxic environment (BW5) of our study. Nevertheless, our results suggest that hypoxia has a non-significant effect on growth during the early growth stage, while, conversely, faster growing tilapia have higher tolerance to hypoxia in the later growing stage, reflected by survival probability. Interestingly, it has been shown that tilapia exposure to a nocturnal hypoxia for 9 weeks led to a better growth performance than normoxia, which is related with a compensatory appetite later in the day [41]. Additionally, Roze et al. [42] has reported that fast growing fish display a better ability to maintain balance to acute hypoxia exposure than slow growing fish, by comparing two genetically different growth strains of Rainbow trout, suggesting a better hypoxia tolerance similar to the findings presented in our study.

In the normoxic environment, six biological processes were significantly enriched for BW1 and BW2, including retinoic acid receptor signalling pathway, apoptotic signalling pathway, liver development, signal transduction, steroid hormone mediated signalling pathway and brain development. Steroid hormone mediated and retinoic acid receptor signalling pathway overlapped with the same stage in the hypoxia environment, which seems mostly involved in general growth and development processes. The overlap in the early growth stage between normoxic and hypoxic environments may result from shared conditions until the first time point. Another possibility is that hypoxia affected small fish less, and there still was sufficient dissolved oxygen as a result of lower overall demand. As fish grew bigger, the metabolic impact of high growth on oxygen consumption and availability may have become more pronounced [43].

For the later growth stage, 12 suggestive SNPs tagging regions containing 22 candidate genes were identified. These included the gene coding for mitochondrial calcium uniporter (*mcu*) that was reported to be a regulator in skeletal muscle growth and homeostasis [44]. The genes coding for oncoprotein-induced transcript 3 (*oit3*) and MAP 6 domain containing 1 (*map 3d1*) were both reported to be related with calcium ion binding activity [45]. Yoshida et al. [19] performed the first genome-wide association study to unravel the genetic architecture of harvest weight in a Nile tilapia population derived from a mixture of the 8th generation GIFT and the wild strains from Egypt and Kenya. In that study, four regions were identified that were significantly associated with harvest weight in LG12, 15, 18 and 22, respectively. However, the genes lying in these regions were not significant in our study. One of the reasons could be that the GIFT population has been selected on growth for many generations and those regions have become fixed. This could also explain the limited number of significant SNPs and candidate genes for growth observed in our study. However, it is also likely the specific variants found by Yoshida et al. were never present in our population to begin with.

The results from the meta-analysis show that five genes play a major role in growth and development during the early growth stage, namely *raraa*, *rarab*, *med24*, *brms11a* and *prpf38b*. Two of them (*raraa*, *rarab*) also showed significance for single GWAS in the normoxic and hypoxic environments, respectively. *Prpf38b* only showed a major effect in the hypoxic environment. The orthologues of this gene in human, zebrafish and mouse have been shown to have a function in the central nerve system [46]. Development related genes found in single GWAS, such as *raraa*, *rarab*, and *med24* were significantly associated in the meta-analysis during the later stages. Nucleotide-binding protein 2 (*nump2*) was reported to be associated with both *IGF1* and *IGFP*3 in a human GWA study [47]. Those results suggest that a few major QTLs determine much of the growth rate. Even though growth rate is known to be determined by many genes [48], similarly in human [49] and cattle [50], it was found that a few genes were exceptionally important in explaining genetic variance.

Moreover, no pathway related to hypoxia tolerance was found in meta-analysis GWAS, which indicates some genes affect body weight in the hypoxic environment while different subset of genes are important for body weight under the normoxic environment (see in Fig. 4a and b). This indicates genotype-by-environment interaction (GxE). However, a GxE analysis for growth rate in the normoxic versus hypoxic environment, based on a quantitative genetic analysis using a genomic relationship matrix derived from the genotyping dataset, showed that the genetic correlation was close to 0.8 [51]. This value suggests some degree of GxE and some reranking of genotypes. Furthermore, there was a large difference in body weight and its variance between environments, which suggests scaling GxE. The genetic correlation of 0.8 suggests that most fish that grow well in a normoxic environment, are also able to grow well in an environment where they experience nocturnal hypoxia. After all, Nile tilapia is a fresh water fish species that has evolved in environments where hypoxia (e.g. as a result of high temperatures, algal blooms or drought) are nocturnal events. Natural selection would favour animals that would be able to cope with these environments if larger fish would have higher reproductive success.

## Conclusions

Clear associations between genotype and growth were found for both hypoxic and normoxic environments. The associated SNPs, and hence the underlying genomic architecture, however, changed over the growing period. Furthermore, the meta-analysis GWAS across two environments suggested that growth was not under the control by the same genes compared to single environmental GWAS, which we interpret as a genotype-by-environment interaction. The functional annotation confirms that hypoxic stress pathways such as MAPK signalling pathway and VEGF signalling pathway play an important role during the later growth stage in the hypoxic environment. Our findings reveal the genetic complexity of body weight gain under a variety of dissolved oxygen conditions in Nile tilapia, and provide an essential insight into how hypoxia affects body weight gain during the growth stage, which will benefit future tilapia breeding programmes in the context of genomic architecture.

## Methods

### Animal resource

The fish were derived from the Aquaculture Extension Centre of the Malaysian Department of Fisheries at Jitra, Kedah State, Malaysia (6°15′32°N; 100°25′47°E). Genetically Improved Farmed Tilapia (GIFT) strain was used in this experiment, and it had been selected for growth based on estimating breeding value (EBV) of harvest weight, with the genetic gain ranged from 5 to 15% per generation. The mate allocation strategy has controlled inbreeding and maintained effective population size [52]. The experimental fish were produced using 72 males from 56 families and 200 females from 73 families (total 81 unique families) of selection line of GIFT generation 15. From each family, fish with EBV for growth that were close to the family mean EBV were selected as a breeder. The experimental fish were mass produced in four hapas (net-enclosures, each 30m^2^) installed in a 500m^2^ earthen pond, aerated by a paddlewheel. For each hapa, 18 male and 50 female breeders were distributed for stocking, and they were removed from mating hapa after 15 days. Fry were reared in the same hapas for 60 days until they reached a taggable size. The fingerlings from each rearing hapa were tagged and then transferred into two earthen ponds with an equal number. Overall 1570 fish were reared in each pond with stocking density of 3 fish/m2. We managed two ponds with the same feeding management (i.e. feeding frequency twice per day, feeding rate was adjusted with fish number), while aeration was the only different treatment between two ponds.

We measured DO every 2 h for 24 h with a total of 7 days during the different grow-out periods using EcoSense® DO200A. The average DO measurements for aerated pond (normoxia) and non-aerated pond (nocturnal hypoxia) are shown in Supplementary Figure 1 and Supplementary Table 1. Both ponds were normoxic (5 mg/L) from 13:00 to 19:00. Non-aerated pond became hypoxic (under 3 mg/L) between 21:00 to next day 9:00. Body weight was measured at five time points (stocking, 55/56 days, 104/105 days, 167/168 days and 217/218 days) growing out in the hypoxic and normoxic environments, respectively. Fish were euthanized using clove oil at a dose of 400 ppm after the experiment. Fin clips were preserved in 95% ethanol and stored at − 20 °C until DNA extraction. More details about this experiment can be found in Mengistu et al. [51].

### Genotyping, variant calling and quality control

DNA extraction and genotyping procedures were described in previous study by Mengistu et al. [51]. In short, we isolated genomic DNA from tilapia fin clips using the DNeasy Blood and Tissue kit. DNA quality was assessed by 260/280 and 260/230 ratios on NanoDrop 2000 spectrophotometer. DNA concentration was measured with Qubit 2.0 Fluorometer. DNA samples were digested with ApeKI, and polymerase chain reaction (PCR) was used to amplify fragments varied from 170 to 350 bp. The prepared libraries were sequenced on the Illumina HiSeq 2000 platform.

Raw sequence reads were trimmed for adaptors and low quality bases with Sickle (https://github.com/najoshi/sickle). The quality of each individual was evaluated by FastQC (version 1.6) [53]. Sequence mapping for 2171 individuals was performed using bwa -mem algorithm [54] aligning to the tilapia reference genome (GenBank accession GCF_001858045.1). Variant calling was analysed with FreeBayes (version 1.0.2) [55] in a default setting excepted these parameters: --min-base-quality 10, −-haplotype-length 0 and --ploidy 2. The SNP data was further filtered by Plink (version1.9) [56] with the following exclusion criteria: Minor Allele Frequency < 2%, genotyping call-rate for SNPs < 80% and individual rate < 70%. Finally, a total of 2063 individuals and 27,090 SNPs were used for subsequent analyses.

### Statistic description, population structure and association analysis

Basic statistics of phenotype data was analysed in R (version 3.5.3). Body weight in our study is not completely following a normal distribution as estimated by Shapiro-Wilk test [57]. Therefore, we compare two paired groups at five time point using the Wilcoxon test. The phenotypic correlation was calculated by spearman’s rank correlation coefficient method. Then, body weight was transformed to better fit the normal distribution by square root method [58]. To estimate the influence of factors such as hapa (early rearing environment) and sex in our experiment, they were tested in a linear model using Stepwise Algorithm [59] with the formula: *y*_*ij*_ = *u* + *α*_*i*_ + *β*_*j*_ + *α*_*i*_ ∗ *β*_*j*_ + *ε*_*ij*_, while *y* is the body weight; *u* is the population mean; *α*_*i*_ is the effect of the i^th^ level of hapa; *β*_*j*_ is the effect of the j^th^ level of sex; ε is the random error effect. It suggested that hapa, sex and their interaction were significant with body weight. Therefore, residuals from the fixed effects model were used for the subsequent association analysis [60].

A principal component analysis (PCA) was performed to estimate population structure before GWAS in Plink (version 1.9) [56]. The top five principal components were added as covariates and included in the subsequent GWAS model as fixed effect to account for the sample structure in this association analysis. Considering the Bonferroni method being overly conservative, we defined the genome-wide significant using the SimpleM method [61]. In total 16,504 independent tests were calculated based on LD (linkage disequilibrium) characteristics. The significant and suggestive lines are 1 and 5% genome-wide significant divided by the SNPs number of independent SNPs in the association. Given the number of effective independent tests, the thresholds for genome-wide and suggestive significance *P*-value were evaluated as 3.03E-06 (0.05/16504) and 6.06E-05 (1/16504), respectively.

A univariate GWAS was performed by implementing a linear mixed model in GEMMA [62]:

$$ \boldsymbol{y}=\boldsymbol{W}\boldsymbol{\alpha } +\boldsymbol{x}\boldsymbol{\beta } +\boldsymbol{\mu} +\boldsymbol{\varepsilon} $$

In this equation, **y** is the a vector of observation on body weight; ***W*** is a covariate matrix of fixed effects (including top five PCs) used to adjust population structure; ***α*** is a vector of the corresponding coefficient including the intercept; ***x*** is a vector of the marker genotypes and ***β*** is the corresponding vector of marker effects for the phenotypes; ***μ*** is a vector of random effects and **ε** is the random residuals. We performed the Wald statistic for each SNP which means we tested the alternative hypothesis *H*_1_: *β* ≠ 0 compared to null hypothesis *H*_*0*_*: β*= 0 for each SNP, which is one of common methods in GWAS studies of quantitative traits [63].

Meta-analysis is powerful to detect shared genetic architecture across traits and populations [64]. Thus, we applied an inverse-variance weighted (IVW) method to estimate the SNP effect and significance combined normoxic and hypoxic environments through Meta (Version 1.7) [65, 66]. The weight (w_i_) for ith environment was calculated by the following equation:

$$ {\mathrm{w}}_{\mathrm{i}}=\frac{1}{{\mathrm{s}}_{\mathrm{i}}^2} $$

Here s_i_ is the standard error of the SNP effect in i^th^ environment GWAS. Then, the effect size and standard error for i^th^ environment GWAS were estimated by the following:

$$ \upbeta =\frac{\sum_{\mathrm{i}=1}^2{\mathrm{w}}_{\mathrm{i}}{\beta}_{\mathrm{i}}}{\sum_{\mathrm{i}=1}^2{\mathrm{w}}_{\mathrm{i}}} $$

$$ {\mathrm{s}}^2=\frac{1}{\sum_{\mathrm{i}=1}^2{\mathrm{w}}_{\mathrm{i}}} $$

The statistical significance was estimated by a z-score of IVW as bellow:

$$ \mathrm{z}=\frac{\upbeta}{\mathrm{s}}=\frac{\sum_{\mathrm{i}=1}^2{\mathrm{w}}_{\mathrm{i}}{\beta}_{\mathrm{i}}}{\sqrt{\sum_{\mathrm{i}=1}^2{\mathrm{w}}_{\mathrm{i}}}} $$

### Post-GWAS analysis

Manhattan and quantile-quantile (Q-Q) plots were generated through the “qqman” package (https://cran.r-project.org/web/packages/qqman/). The inflation factor λ was calculated to indicate the influence of population structure in the association analyses. Candidate regions were defined as the genomic regions that located 20 kb upstream and downstream of the genome-wide significant SNPs. In order to identify candidate genes nearby the significant SNPs, we used the Custom Annotations function to create an annotation set with parameters (−-distance 20,000 --gene_phenotype --symbol) in Ensembl Variant Effect Predictor (VEP) [67]. All protein sequences of candidate genes were extracted through reference protein sequence with an inhouse python script, and were further used for functional enrichment analysis in STRING V11.0 [68]. The false discovery rate (FDR) adjusted *p*-value of 0.05 was used to define significant enrichment.

## Supplementary Information

**Additional file 1: Supplementary Figure 1.** Variation of dissolved oxygen in the normoxic and hypoxic environments during the 24-h cycle.

**Additional file 2: Supplementary Figure 2.** Body weight comparison amongst four hapas in the normoxic and hypoxic environments.

**Additional file 3: Supplementary Figure 3.** Two-dimensional plots of all individuals using SNP markers in the hypoxic environment.

**Additional file 4: Supplementary Figure 4.** Two-dimensional plots of all individuals using SNP markers in the normoxic environment.

**Additional file 5: Supplementary Figure 5.** Quantile-quantile plots through the whole growth stage in the hypoxic environment.

**Additional file 6: Supplementary Figure 6.** Quantile-quantile plots through the whole growth stage in the normoxic environment.

**Additional file 7: Supplementary Table 1.** Measurements of dissolved oxygen in the normoxic and hypoxic environments.

**Additional file 8: Supplementary Table 2.** Information of genome-wide significant and suggestive SNPs in the hypoxic environment.

**Additional file 9: Supplementary Table 3.** Information of genome-wide significant and suggestive SNPs in the normoxic environment.

**Additional file 10: Supplementary Table 4.** Information of genome-wide significant SNPs across the two environments by meta-analysis.

**Additional file 11: Supplementary Table 5.** Functional enrichment for the early stage in the hypoxic environment.

**Additional file 12: Supplementary Table 6.** Functional enrichment for the early stage in the normoxic environment.

**Additional file 13: Supplementary Table 7.** Functional enrichment for the whole growth stage across the two environments by meta-analysis.

## Data Availability

The genotype and phenotype data generated or analysed during this study are in the Harvard Dataverse repository with accession number KCBEON, which can be accessed at 10.7910/DVN/KCBEON. The tilapia reference genome (GCF_001858045.1_ASM185804v2_genomic.fna.gz) and annotation file (GCF_001858045.1_ASM185804v2_genomic.gff.gz) were downloaded from the NCBI genome assembly website (https://ftp.ncbi.nlm.nih.gov/genomes/all/GCF/001/858/045/GCF_001858045.1_ASM185804v2/). The authors declare that all data supporting the findings are available within this article and its supplementary files.

## Abbreviations

AMPK: AMP-activated protein kinase
AURKA: Aurora kinase A
BW: Body weight
CV: Coefficient of variation
DO: Dissolved oxygen
EBV: Estimated breeding value
EFNA3: Ephrin-A3
FDR: False discovery rate
GIFT: Genetically Improved Farmed Tilapia
GO: Gene ontology
GWAS: Genome Wide Association Study
GxE: Genotype-by-environment interaction
HAS1: Hyaluronan synthase
HIF: Hypoxia-Inducible factors
IVW: Inverse-variance weighted
KEGG: Kyoto Encyclopedia of Genes and Genomes
LD: Linkage disequilibrium
LG: Lineage group
MAPK: Mitogen-activated protein kinase
MAP 3D1: MAP 6 domain containing 1
MCU: Mitochondrial calcium uniporter
MED24: Mediator of RNA polymerase II transcription subunit 24
NUMP2: Nucleotide-binding protein 2
OIT3: Oncoprotein-induced transcript 3
PCA: Principal component analysis
PC: Principal component
PGK1: Phosphoglycerate kinase 1
Q-Q: Quantile-quantile
RORA: Retinoic acid receptor-related orphan receptor alpha
ROS: Reactive oxygen species
SD: Standard deviation
SNP: Single nucleotide polymorphism
TH: Tyrosine hydroxylase
VEGF: Vascular endothelial growth factor
VEP: Variant Effect Predictor
