# Supplementary figures and images for: Genome-wide association analysis of adaptation to oxygen stress in Nile tilapia (Oreochromis niloticus)
Source: BMC Genomics. 2021 Jun 9;22:426. doi: 10.1186/s12864-021-07486-5 (PMC8188787; doi:10.1186/s12864-021-07486-5)

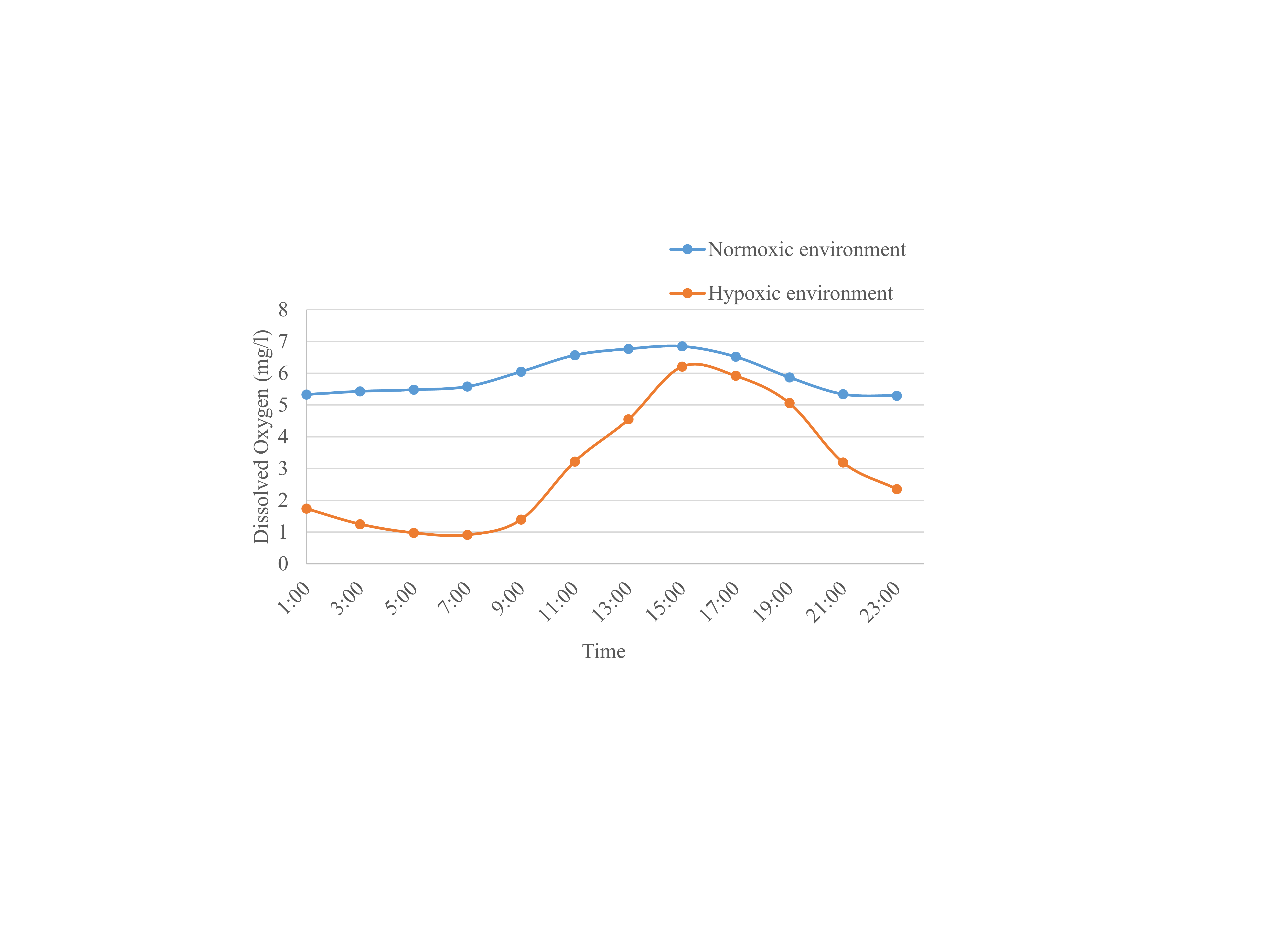

Supplement: Supplementary file 1 — Additional file 1: Supplementary Figure 1. Variation of dissolved oxygen in the normoxic and hypoxic environments during the 24-h cycle. [file 12864_2021_7486_MOESM1_ESM.tif]

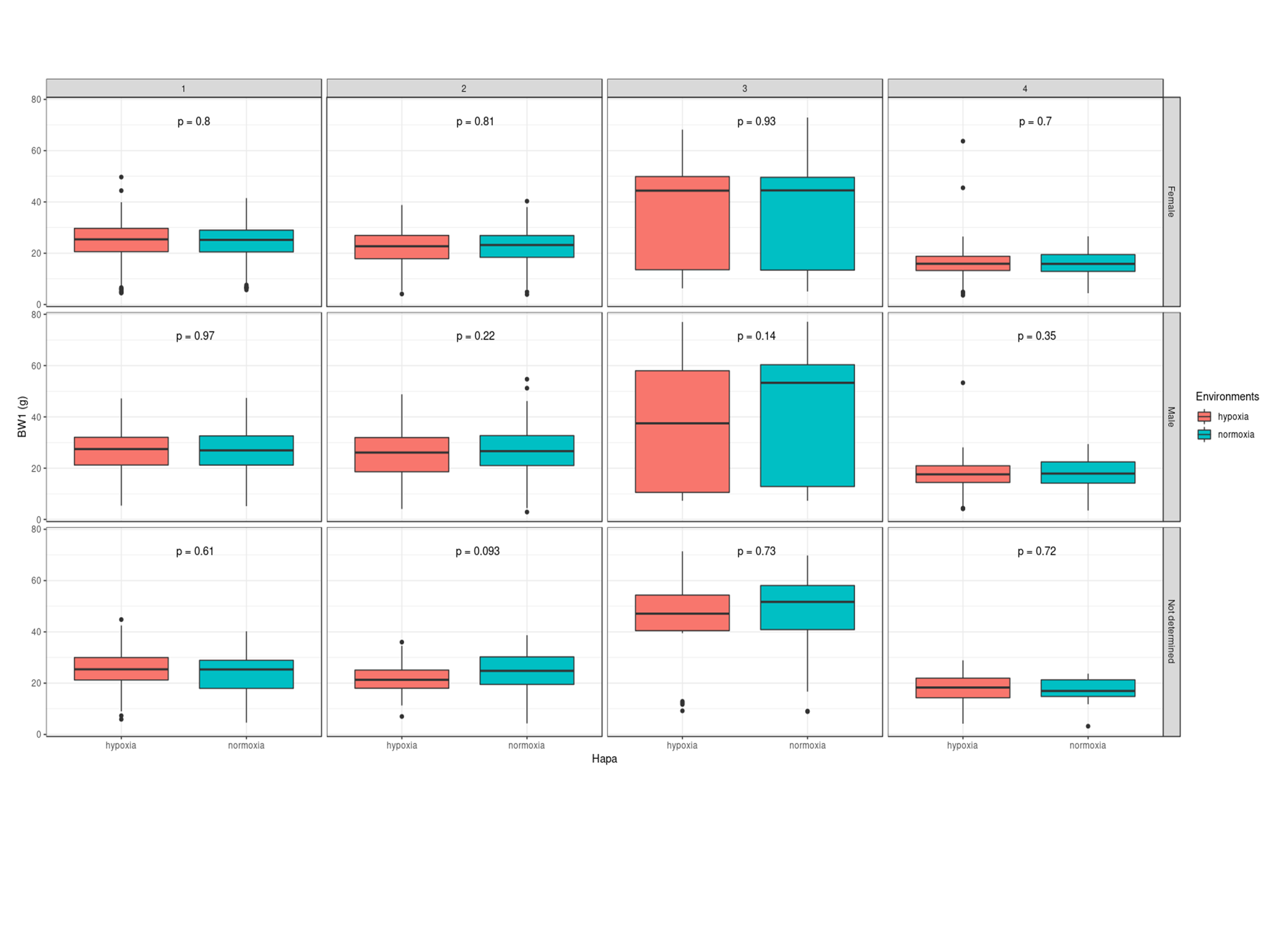

Supplement: Supplementary file 2 — Additional file 2: Supplementary Figure 2. Body weight comparison amongst four hapas in the normoxic and hypoxic environments. [file 12864_2021_7486_MOESM2_ESM.tif]

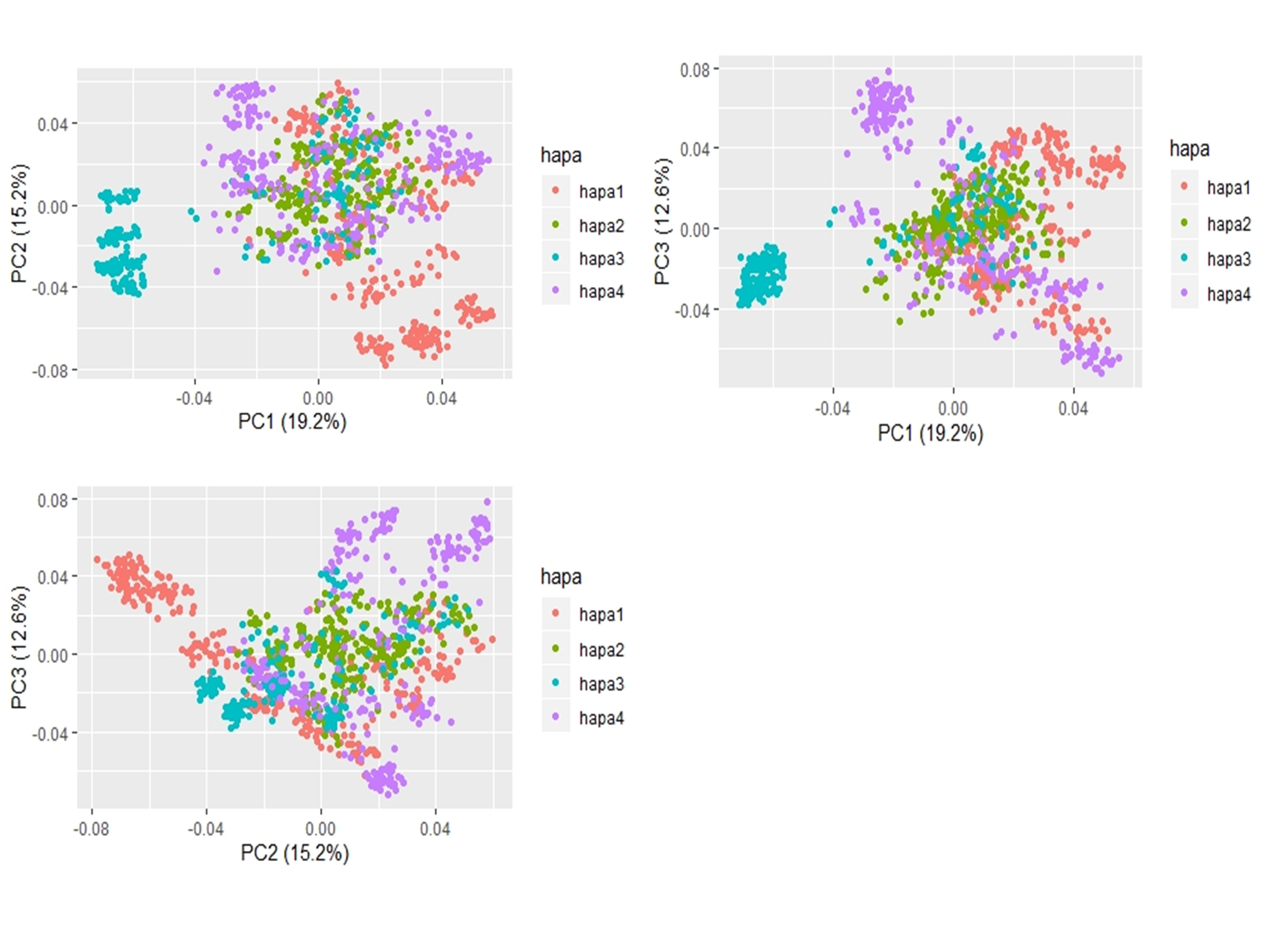

Supplement: Supplementary file 3 — Additional file 3: Supplementary Figure 3. Two-dimensional plots of all individuals using SNP markers in the hypoxic environment. [file 12864_2021_7486_MOESM3_ESM.tif]

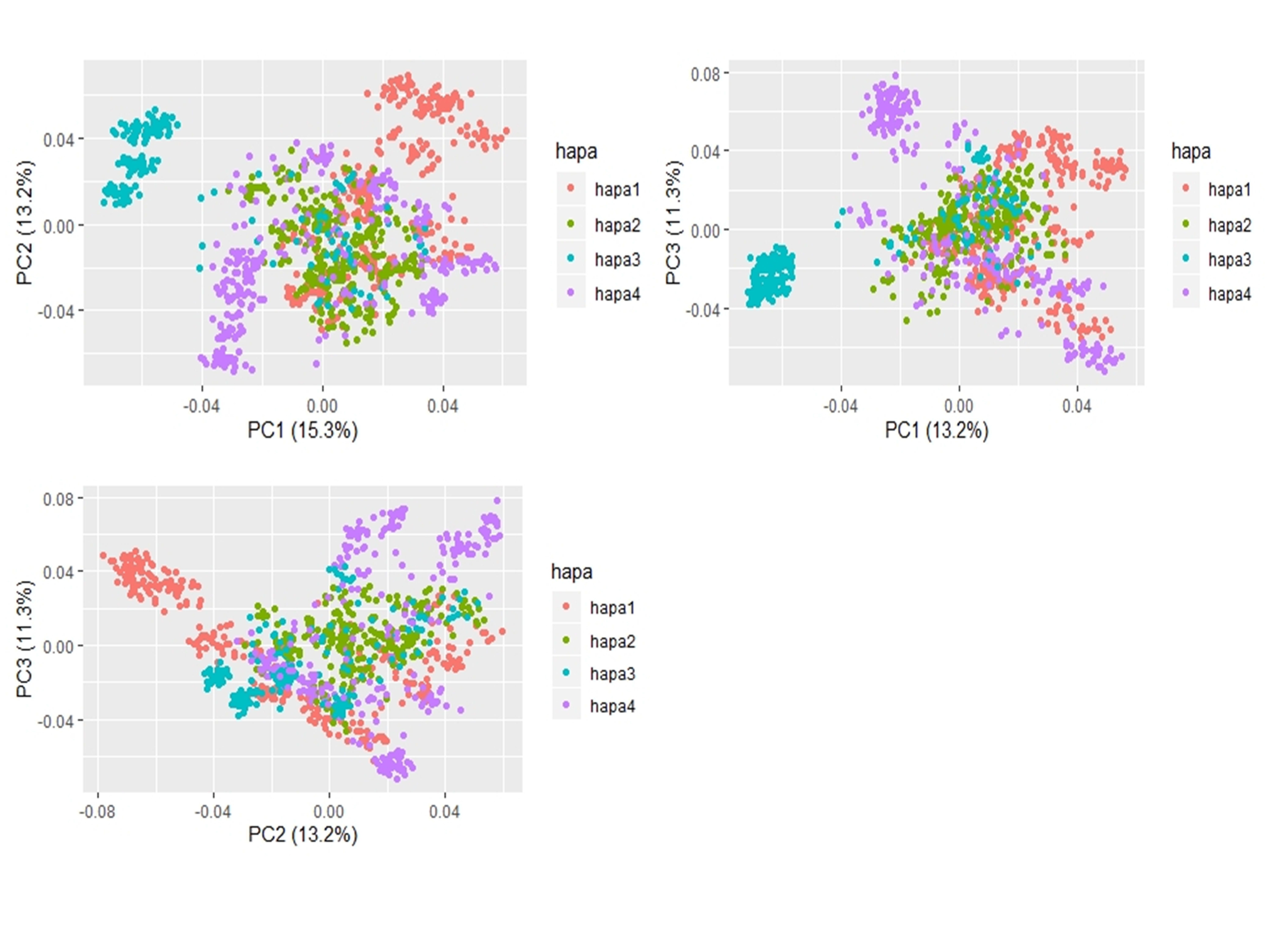

Supplement: Supplementary file 4 — Additional file 4: Supplementary Figure 4. Two-dimensional plots of all individuals using SNP markers in the normoxic environment. [file 12864_2021_7486_MOESM4_ESM.tif]

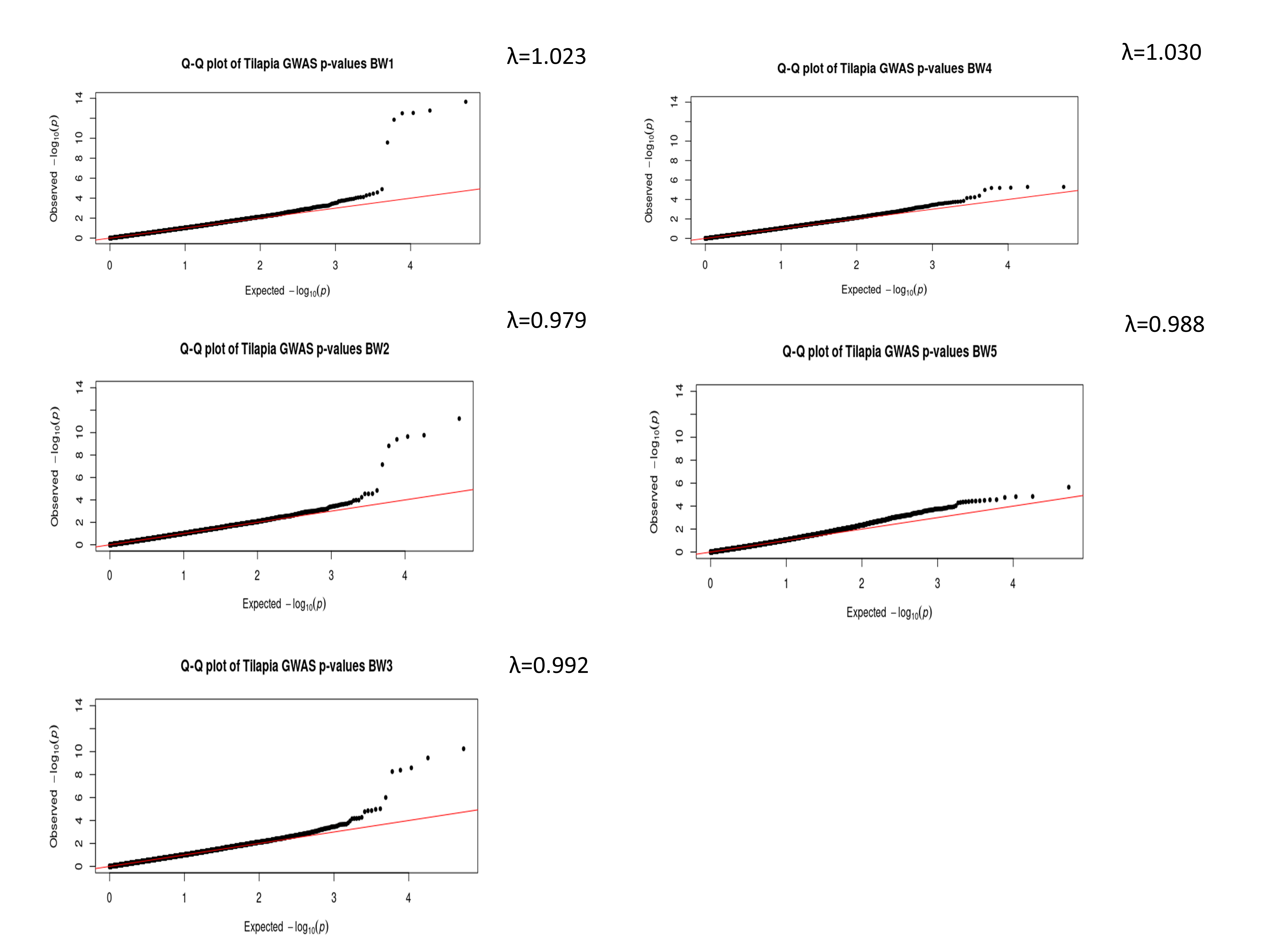

Supplement: Supplementary file 5 — Additional file 5: Supplementary Figure 5. Quantile-quantile plots through the whole growth stage in the hypoxic environment. [file 12864_2021_7486_MOESM5_ESM.tif]

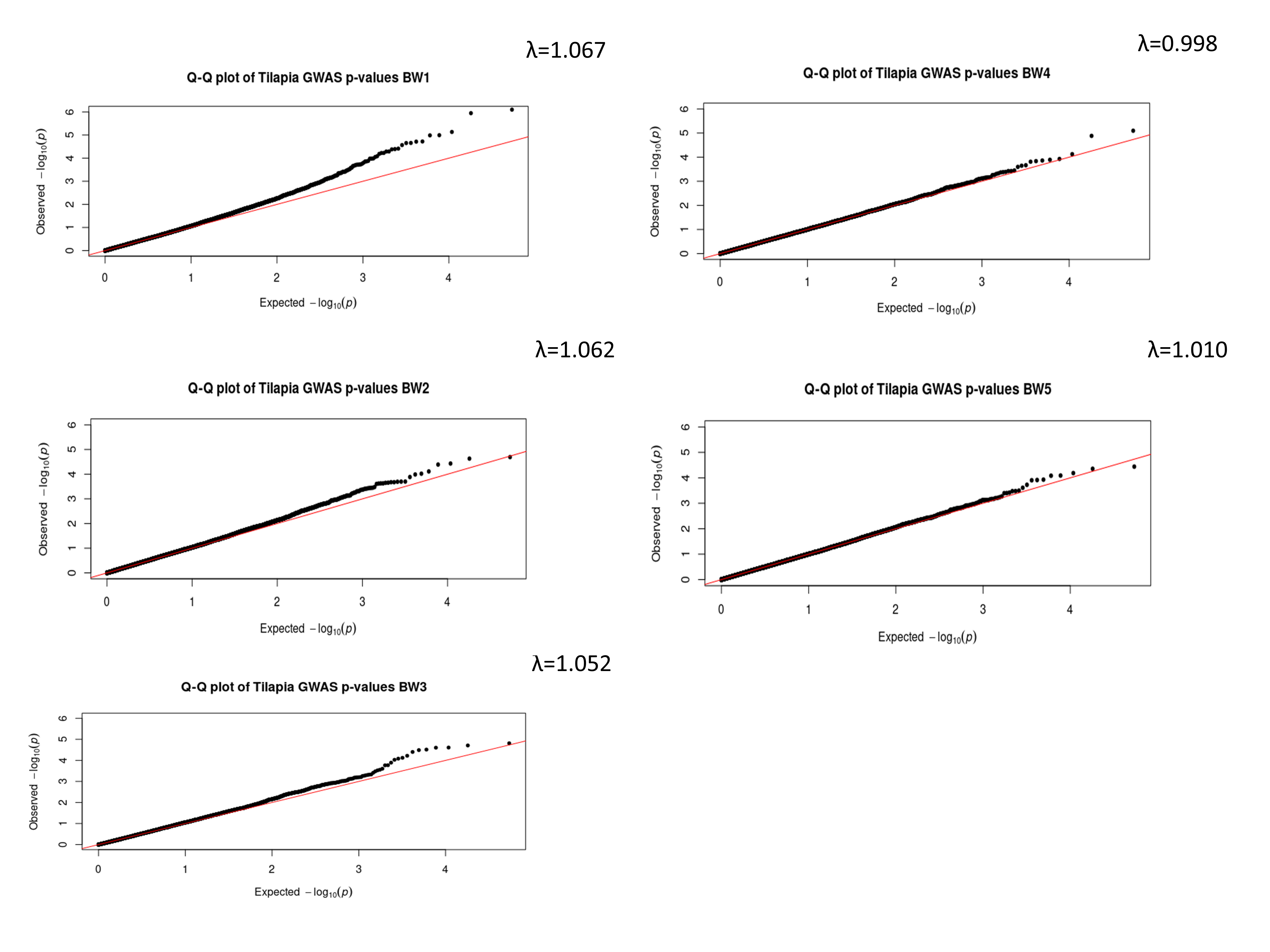

Supplement: Supplementary file 6 — Additional file 6: Supplementary Figure 6. Quantile-quantile plots through the whole growth stage in the normoxic environment. [file 12864_2021_7486_MOESM6_ESM.tif]
